# MS275 as Class I HDAC inhibitor displayed therapeutic potential on malignant ascites by iTRAQ-based quantitative proteomic analysis

**DOI:** 10.1186/s12876-022-02101-7

**Published:** 2022-01-21

**Authors:** Li Du, Dongyuan Wang, Xiuqi Wei, Chang Liu, Zhuanglong Xiao, Wei Qian, Yuhu Song, Xiaohua Hou

**Affiliations:** 1 Division of Gastroenterology, Union Hospital, Tongji Medical College, Huazhong University of Science and Technology, Wuhan, 430022 People’s Republic of China; 2 Department of Pharmacy, Union Hospital, Tongji Medical College, Huazhong University of Science and Technology, Wuhan, 430022 People’s Republic of China; 3 Department of Clinical Laboratory, Union Hospital, Tongji Medical College, Huazhong University of Science and Technology, Wuhan, 430022 People’s Republic of China

**Keywords:** Histone deacetylase inhibitor, MS275, CDK4, Malignant ascites, Proteomic, Cell cycle arrest, Apoptosis

## Abstract

**Background:**

Malignant ascites is a manifestation of end stage events in a variety of cancers and is associated with significant morbidity. Epigenetic modulators play a key role in cancer initiation and progression, among which histone deacetylases (HDACs) are considered as one of the most important regulators for various cancer development, such as liver cancer, ovarian cancer, and pancreatic cancer et al. Thus, in this paper, we sought to explore the therapeutic effect of HDAC inhibitor on malignant ascites.

**Methods:**

In this report, we tested the therapeutic effect of different isoform selective HDAC inhibitors (Class I HDACI MS275, Class IIa HDACI MC1568, pan-HDAC inhibitors SAHA) on malignant ascites in vitro and in vivo. We further used proteome analysis to find the potential mechanisms for malignant ascites therapy.

**Results:**

Among the different isoform-selective HDAC inhibitors, the class I selective HDACI, MS275, exhibited preferential inhibition on various ascites cells. MS275 could induce cell cycle arrest in G0/G1 phase and promote apoptosis on ascites cells. Through proteome analysis, we found MS275 could downregulate proteins related to cell cycle progression, such as CDK4, CDC20, CCND1; MS275 could upregulate pro-apoptosis proteins such as PAPR1, LMNB2 and AIFM1; in addition, MS275 could change the expression of tumorigenic proteins related to the specific malignant ascites bearing tumors, such as TSP1 and CDK4 for bladder cancer. We then confirmed that abemaciclib (CDK4/6 selective inhibitor) could inhibit the proliferation of ascites cells, and the combination of abemaciclib and MS275 had synergistic anti-tumor effect. Finally, we found that MS275 could in vivo inhibit malignant ascites progression (ascites volume: 2.9 ± 1.0 mL vs 7.5 ± 1.2 mL, *p* < 0.01), tumor growth, and prolong 66% of the life-span when compared with the untreated group.

**Conclusion:**

This present research revealed that the class I selective HDAC inhibitor, MS275, could effectively inhibit malignant ascites development and tumor growth via multiple pathways. These results indicated that HDACI could have great potential for clinical therapy of malignant ascites.

## Background

Ascites is the pathological accumulation of fluid in the peritoneal cavity [1]. Malignant ascites occurs for ~ 10% of all cases of ascites, and is a manifestation of end stage events in a variety of cancers such as: liver, colon, gastric, pancreatic, ovarian, renal, bladder, breast, melanoma, etc [2, 3]. It is known that about 50% of patients with malignant ascites present with ascites at the initial diagnosis of their cancer [4]. The onset and progression of malignant ascites is associated with deterioration in quality of life (QoL) and significant morbidity [4]. There are, however, no generally accepted evidence-based guidelines for the treatment of this condition [4].

Epigenetic alteration is one of the hallmarks in malignant tumors [5, 6]. Histone deacetylases (HDACs), the important epigenetic mediators, can remove acetyl groups from histone lysine residues. HDACs are frequently upregulated in cancers and can silence tumor suppressor genes and apoptosis inducers to promote cancer progression [7]. According to the homology to yeast deacetylases, eighteen HDACs are divided into four groups: class I HDACs (HDAC1-3 and 8), class IIa (HDACs: 4, 5, 7, 9), class IIb (HDACs: 6, 10), class III HDACs (SIRT1-7) and class IV HDAC (HDAC11) [8–10]. Class I, II, IV HDACs are zinc dependent, whereas class III HDACs require cofactor NAD^+^ for their catalytic activity. Due to the important regulation of HDACs in cancer development, HDACs have become one of the promising anti-cancer targets [11]. Nowadays, HDAC inhibitors (HDACIs) are broadly developed and exhibit excellent effect in various types of cancers [7]. Due to the non-specific toxicity of pan-HDAC inhibitors, development of isoform-selective HDAC inhibitors has become the main direction for HDACIs [11]. MS275 (entinostat), the class I selective HDAC inhibitor, have been proved to be effective on various solid tumor (such as renal cancer, breast cancer, melanoma) and hematological malignancies in phase I and II clinical trials [12–18].

Till now there is no report about the effect of HDACs on ascites development, however many reports have indicated that HDAC aberrance promotes the progression of liver cancer, colon cancer, pancreatic cancer, ovarian cancer, etc., which are the common etiologies of malignant ascites. For example, for liver cancer, the aberrant expression of HDACs are reported to be associated with liver cancer proliferation, cell cycle regulation, differentiation, apoptosis, and neo-angiogenesis as well as migration. HDACs are also highly expressed in colon tumors and HDAC inhibitors can influence the growth of colon cancer cells [19]. The aberrant expression of HDACs, especially class I HDAC, are also found in pancreatic cancer. HDACIs induce cell cycle arrest of pancreatic cancer cells in a p53 independent way. Besides, HDACI can inhibit tumor angiogenesis by regulation of hypoxia-inducible factor-1 alpha (HIF-1a) and vascular endothelial growth factor (VEGF), and inhibit pancreatic cancer metastasis by reverse of epithelial to mesenchymal transition (EMT) [8].

Due to the complex sources of MA (malignant ascites) and the limited therapeutic methods, in this paper we investigated the therapeutic effect of HDACI, the broad-spectrum anticancer drug, on malignant ascites and further analysis the anti-tumor mechanism through proteome analysis. This study will provide an alternative option for clinical malignant ascites therapy.

## Materials and methods

### Cell culture

The mice malignant ascites cell line, S180 (purchasing from the Chinese Academy of Science, Shanghai, China), H22 (Chinese Academy of Science, Shanghai, China), and EAC (China Center for Type Culture Collection, Wuhan, China) were cultured in 1640 medium supplemented with 10% fetal bovine serum (Gemini, 900-108, USA), 100 µg/ml streptomycin and 100 µg/ml penicillin (Sigma,V900929,USA). All these cells were maintained in a humidified incubator containing 5% CO2 at 37 °C. Cells growing in the mid-logarithmic growth phase were utilized in all experiments.

### Cell viability assay

Cell viability was measured via cell proliferation test with a Cell Counting Kit-8 (Dojindo, Japan). Cells were seeded into 96-well plates and cultured for 24 h, then treated with different classes of HDACIs for 48-h incubation. The HDACIs were dissolved in 1640 medium (10%FBS) containing 0.4% (v/v) dimethyl sulfoxide (DMSO), and the control group were treated with 0.4% (v/v) DMSO alone. 10 μL CCK8 reagent was added to 96-well plates and incubated for another 3 h. The absorbance (OD450 nm) was measured using a microplate reader (TECAN, Switzerland) and then analyzed by Graphpad prism 7.

### Cell cycle analysis

As for the cell cycle arrest experiments, cells were seeded in a 24-well plate and cultured for 24 h, then treated with MS275 in 0 μM, 2.5 μM and 5 μM for 24 h incubation. Each well contained 0.4% (v/v) DMSO. Then, cells were fixed with 70% ethanol overnight, suspended in PBS with 0.3% Triton at 37C for 10 min, then stained with 500 μL PI kit (BD Pharmingen, 550825, USA) for 15 min. The samples were analyzed by flow cytometry (LSRFortessa, BD, USA) and the percentages of cells in G0/G1, S, and G2/M phases were analyzed.

### Apoptosis assay

The apoptosis assay was performed according to the manufacturer's instructions using an Annexin V/FITC-PI Apoptosis Detection Kit I (Beyotime C1062, Shanghai, China). Briefly, cells were seeded in a 24-well plate and cultured for 24 h, then treated with MS275 in 0 μM, 2.5 μM, 5 μM and 10 μM respectively for 36-h incubation. Each well contained 0.4% (v/v) DMSO. The cells were harvested and treated with FITC-labeled Annexin V and propidium iodide (PI) as the protocol indicated. Cells that showed Annexin V-positive were in early stages of apoptosis, both Annexin V and PI-positive were in the late stage, while normal cells showed both Annexin V and PI-negative.

### Protein quantification

The S180 cells were treated with 5 μM MS275 or 0.4% (v/v) DMSO as control group for 48-h incubation and the sufficient samples were submitted for iTRAQ quantitative proteomics analysis (BGI, Shenzhen, China). Protein extraction, SDS-PAGE purification, protein digestion and peptide quantification was dealt as reference [1]. The peptide samples were respectively labeled using the iTRAQ Reagent-4plex Multiplex Kit (AB SCIEX) according to the manufacturer's instructions. The labeled peptide fractionation was carried out by using Shimadzu LC-20 AB liquid phase system with 5  μm 4.6 × 250 mm Gemini C18 column and followed by HPLC (Thermo UltiMate 3000 UHPLC). The nanoliter liquid phase separation end was directly connected to the mass spectrometer with a tandem mass spectrometer Q-Exactive HF X (Thermo Fisher Scientific, San Jose, CA). The MS/MS data were searched against the Mascot database (uniprot-human 20151227.fasta) for peptide identification and quantification. The searching result of peptides was filtered by FDR *p* value with a cut off of 0.05. Based on statistical dispersion of the dataset, ratio of > 1.5 or < 0.667 was used as a strict significance cutoff to acquire a short list of the differentially distributed proteins as indicated in the data legends.

### Animals

C57BL/6J female mice (weight, 17–22 g) were purchased from Beijing Vital River Laboratory Animal Technology, Beijing, China. Mice were maintained at a temperature of 23 ± 2 °C and a relative humidity of 50 ± 10%, with 12 h light/dark cycles. All experiments were conducted and approved by the Tongji Medical School Animal Care for Laboratory Animals, Huazhong University of Science and Technology, Wuhan, China.

### Experimental animal grouping and administration

5 × 10^6^ cells/ml of S180 were suspended in 1640 medium, and 0.2 ml of the suspension was injected into the peritoneal cavity of each mouse. In total, we selected 30 mice with abdominal bulging from 40 mice in total at the 8th day after intraperitoneal inoculation. Mice were equably divided into 2 groups (n = 15 for each group): the treatment group were treated with MS275 (50 mg/Kg, Selleck Chemicals) every other day for 4 consecutive intraperitoneal injection, while the control group were treated with DMSO. Five mice in each group were selected randomly, and the body weight was measured every day, the peritoneal tumors were observed and the volume of ascites were determined when sacrificing mice by cervical dislocation at 24 h following the final treatment. The remaining 10 mice in each group were recorded for 30 days after tumor cells transplantation for survival analysis, and the increase in life span was calculated according to the following formula: Increase in life span (%) = (T/C − 1) × 100, where T represented the average survival (days) of mice in MS275 treatment group, and C represented the average survival (days) of mice in the DMSO treated control group.

### Statistical analysis

Experiments were repeated at least three times. All statistical analyses were conducted using the statistical software SPSS (version 23.0; IBM, Armonk, New York) with *p* < 0.05 considered statistically significant. Data are expressed as the mean ± standard deviation. A Student's t-test was used to analyze differences between two groups.

## Results

### HDACI inhibited proliferation of ascites cells

**Fig. 1:**
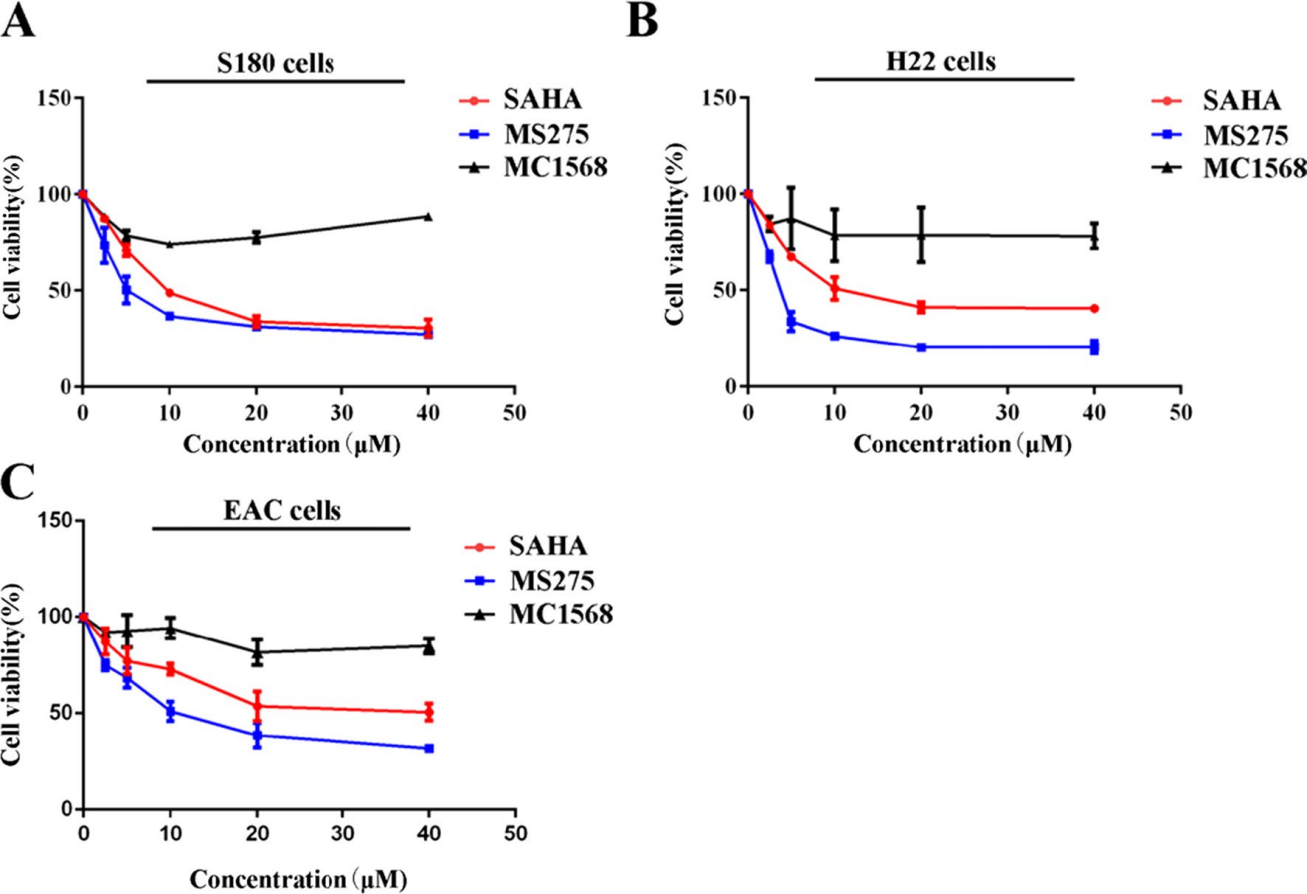
Viability of S180, H22 and EAC cells treated with HDACIs**.** S180 (**A**), H22 (**B**) and EAC cells (**C**) were incubated with 0 μM, 2.5 μM, 5 μM, 10 μM, 20 μM, 40 μM SAHA, MS275 or MC1568 for 48 h. Error bars represent SEMs of at least three independent measurements

To study the selective toxicity of different isoform-selective HDACIs on ascites cells, the class I selective HDACI MS275, the class II selective HDACI MC1568 and the broad-spectrum HDACI SAHA were chosen to test the cytotoxicity to ascites cells. Because primary peritoneal carcinoma is rare [20, 21] and malignant ascites are mainly originated from different metastatic peritoneal carcinoma, here we chose 3 different ascites cell. S180, H22 and EAC are murine sarcoma cancer cell, murine hepatocarcinoma cell, and murine mammary adenocarcinoma cell respectively, and the related ascites model induced by these cells are commonly used [21, 22]. As shown in Fig. 1A–C, MS275 showed better anti-tumor effect than SAHA or MC1568, with an IC50 of 6.5 ± 1.2 μM in S180 cells, 3.7 ± 0.4 μM in H22 cells and 12.1 ± 1.6 μM in EAC cells. While SAHA had an IC50 of 12.0 ± 0.6 μM in S180 cells, 15.1 ± 0.1 μM in H22 cells and 34.0 ± 7.6 μM in EAC cells. In summary, the class I selective HDACI, MS275, exhibited preferential inhibition on different ascites cells.

### MS275 induced cell cycle arrest and apoptosis on malignant ascites cells

**Fig. 2:**
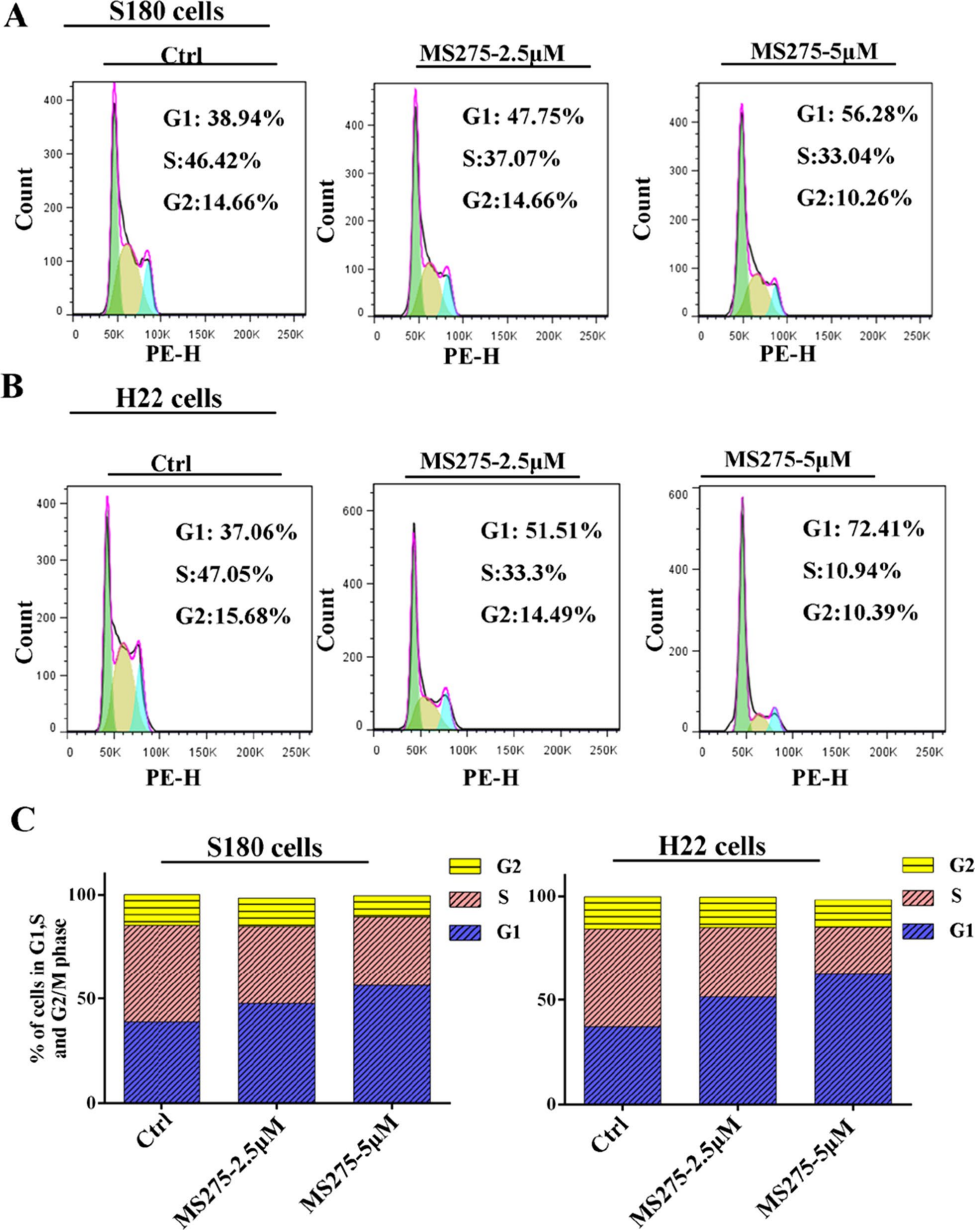
Cell cycle analysis of MS275 treated cancer cells. Percentage of S180 (**A**, **C**) and H22 (**B**, **C**) cells in each mitotic phase after treatment with 0 μM, 2.5 μM, 5 μM MS275 for 24 h. Three independent measurements are repeated, and mean value is presented in (**C**)

**Fig. 3:**
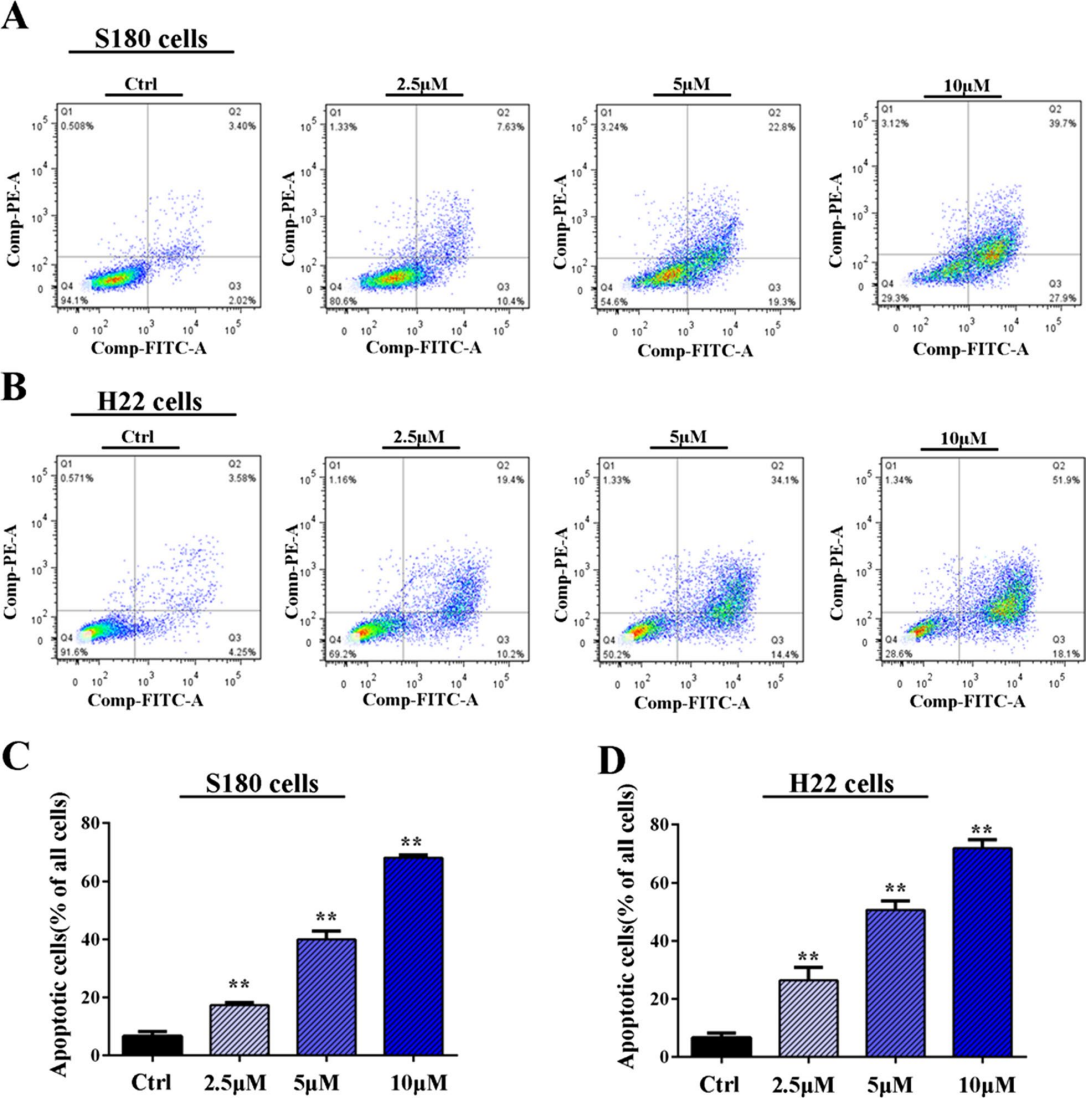
Apoptosis analysis of MS275 treated cancer cells. FITC-Annexin V and propidium iodide (PI) cell apoptotic assay of S180 (**A**, **C**) and H22 (**B**, **D**) cells after treatment with 0 μM, 2.5 μM, 5 μM, 10 μM MS275 for 36 h. The number of apoptotic cells stained with Annexin V/PI was measured by flow cytometry. Late apoptotic cell counts in the upper right quadrant (Q2) and early apoptotic cell counts in lower right quadrant (Q3) for different treatment groups. Apoptosis cell percentage in (**C**) equal s the sum of Q2 and Q3. **, *P* < 0.01 versus control group, Error bars represent SEMs of at least three independent measurements

To study the alteration of cell cycle distribution of MS275 on ascites cells, FACS assays were performed as shown in Fig. 2. We found MS275 displayed a significant cell cycle arrest in G0/G1 phase with dose-dependent manner. The aberrance of HDACs is reported to be associated with cellular functions such as cell apoptosis. To verify if MS275 could induce cell apoptosis for ascites cells, Annexin V/PI assay was performed. For S180 cell, MS275 could induce 17.4% cell apoptosis when treated with 2.5 μM MS275 and the percentage increased to more than 50% when treated with 10 μM MS275 shown in Fig. 3A, C. For H22 cell, MS275 could induce about 26.5% cell apoptosis when treated with 2.5 μM MS275 and the percentage increased to more than 50% when treated with 10 μM MS275 shown in Fig. 3B, D. To sum up, MS275 could significantly induce cell apoptosis in ascites cells S180 and H22 in dose-dependent manner.

### MS275 influenced protein distribution

**Fig. 4:**
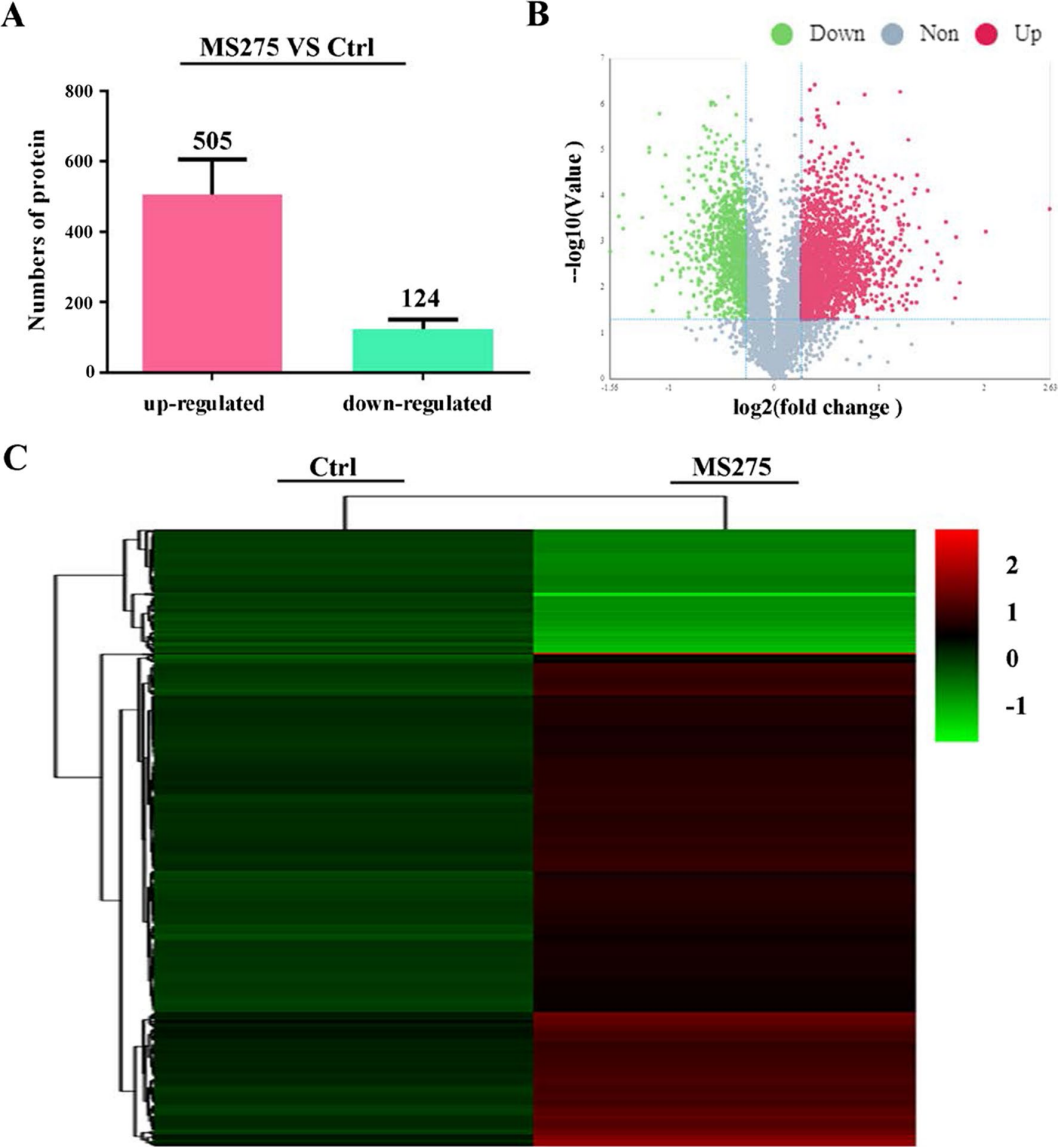
Proteomic analysis of S180 cells treated with DMSO or 5 μM MS275 respectively for 48 h. **A** Proteomic analysis of upregulated and downregulated protein in S180 cells treated with DMSO or MS275. **B** Volcano plots showing differentially distributed proteins comparing DMSO control and MS275 treated group. Proteins represented by red dots indicate upregulation, and green dots indicate downregulated proteins (*p* < 0.05, ratio > 1.5 or < 0.667). **C** Hierarchical clustering of the differentially distributed proteins identified from each group (ratio > 1.5 or < 0.667). The color scale indicates the expression level of each protein across the two groups

To investigate the potential role of proteins in ascites cells, we distinguished the proteomic profiles of MS275 treated S180 cells and untreated cells by iTRAQ analysis. In total, 629 differentially distributed proteins were identified, of which 505 proteins were upregulated and 124 were downregulated (Fold change > 1.5 or < 0.667, t-test *p* value < 0.05) (Fig. 4A). The distribution of significantly changed proteins was illustrated in a volcano plot shown in Fig. 4B (Fold change > 1.5 or < 0.667, t-test *p* value < 0.05), and the expression levels of all proteins in each sample category were visualized in a hierarchical clustering heatmap shown in Fig. 4C (Fold change > 1.5 or < 0.667, t-test *p* value < 0.05). The results indicated that MS275 could significantly influence the expression of proteins in ascites cells.

### MS275 inhibited ascites cell proliferation through regulation of cell cycle, apoptosis and tumorigenic pathways

To explore the underlying mechanism of MS275 on ascites cell proliferation, we conducted iTRAQ analysis to compare the proteomic difference between MS275 treated and untreated S180 cells. Overall, MS275 caused dramatic changes of protein expression in S180 cells. Proteins related to cell cycle progression, such as CDC20, CDK4, CCND1 [23–28] were more abundantly expressed in the controls groups than the treated S180 cells (Table 1), suggesting that MS275 affected the checkpoint passing of cycle phase, which was also consistent with our cycle arrest assay result. Proteins related to necroptosis, apoptosis and ferroptosis had higher expression in MS275 treated S180 cells. Specifically, PARP1, H2AX, VDAC1 and STAT2 could promote necroptosis [29–32]; PAPR1, LMNB2 and AIFM1 could promote apoptosis [33–35]; HMOX1 could promote ferroptosis [36]. This was also supported by our cell apoptosis assay data. Simultaneously this point might be reinforced by upregulation of p53 target proteins. Proteome analysis also indicated that MS275 could promote S180 cell senescence, which was characterized by higher expression of pro-senescence proteins, such as VDAC1, VDAC3 [37, 38], and lower expression of anti- senescence proteins such as CDK4, CCND1 [39]. Finally, we showed the specific protein alterations related to the different malignant ascites bearing tumors development, such as TSP1 and CDK4 for bladder cancer, RALA and RALB for pancreatic cancer, GSTM6 and HMOX1 for hepatocellular cancer, etc. In addition, the protein expressions of various tumors caused by chemical carcinogenesis, viral carcinogenesis also changed in the MS275 treatment group. Although it was not the same cancer model as our experiment, it may still hint the possible tumor related pathway changes caused by MS275 (shown in Table 1).

**Table 1:** Proteins related to cell growth and death, replication and repair, and cancers

Pathway level	Pathway	Related proteins
Cell growth and death	Cell cycle	SFXN1;CCND3; SFXN2;
		*BUB1B;CDC20;EP300;CDK4;HIC2;RBX1;CCND1*
	Apoptosis	PARP1;LMNB1;LMNB2;TR10B;ENSA;ITPR3;AIFM1;CCD51;CATL1
	Necroptosis	PARP1;H2AX;VDAC1;VDAC3;TR10B; AIFM1; STAT2; ADT4; ADT1; H2AY; VDAC2; *PPID*
	Ferroptosis	STEA3;ACSL3;VDAC3;HMOX1;4F2;ACSL1; VDAC2; MBOA5
	Cellular senescence	CCND3;VDAC1;Z36L3;VDAC3;ITPR3;ISC2A;HA1D;ADT4;ADT1;VDAC2;*PPID;HUS1;CDK4;CCND1*
	p53 signaling pathway	STEA3;CCND3;TSP1; ZN346; *CDK4;CCND1*
Replication and repair	Nucleotide excision repair	*DPOE4;RBX1*
	Base excision repair	PARP1;*DPOE4*
	DNA replication	SFXN1;SFXN2; *DPOE4*
	Homologous recombination	*XLR*
Cancers: Overview	Chemical carcinogenesis	GSTM6;GSTM7;PGH2;HYEP;MGST3;U2AFM
	Viral carcinogenesis	ATF2;SCRIB;VDAC3;H2B2B;HA1D;LRRC1;CRLF1;VA0D1;CCND3;LR*C58;H4;HDAC7;CDC20;EP300;CDK4;RANG;CCND1*
	MicroRNAs in cancer	DIK2A;VIME;PGH2;IF2B1;ENSA;TSP1;HMOX1;*EP300;CCND1*
	Transcriptional misregulation in cancer	MLF1;PBX3;K319L;CEBPB;MITF;TNR16;*Q9JLZ6*
	Proteoglycans in cancer	SDC4;ESPN;TSP1;ITPR3;ITB5;FINC;ITAV;;CATL1;|CD63;*CCND1*
	Choline metabolism in cancer	DGKE;CTL2;CHKA
	Central carbon metabolism in cancer	GTR1
Cancers: Specific types	Bladder cancer	TSP1;*CDK4;CCND1*
	Pancreatic cancer	RALA;RALB;*CDK4;CCND1*
	Renal cell carcinoma	GTR1;*EP300;RBX1*
	Colorectal cancer	RALA;RALB;*CCND1*
	Hepatocellular carcinoma	GSTM6;GSTM7;MGST3;HMOX1;*CDK4;CCND1*
	Endometrial cancer	*CCND1*
	Gastric cancer	*CCND1*

MS275 treatment versus Ctrl, *p* < 0.05

Upright letters = upregulated; inclined letters = downregulated

### CDK4 inhibitor and Class I HDACI had synergistic effect to suppress the proliferation of ascites cells

**Fig. 5:**
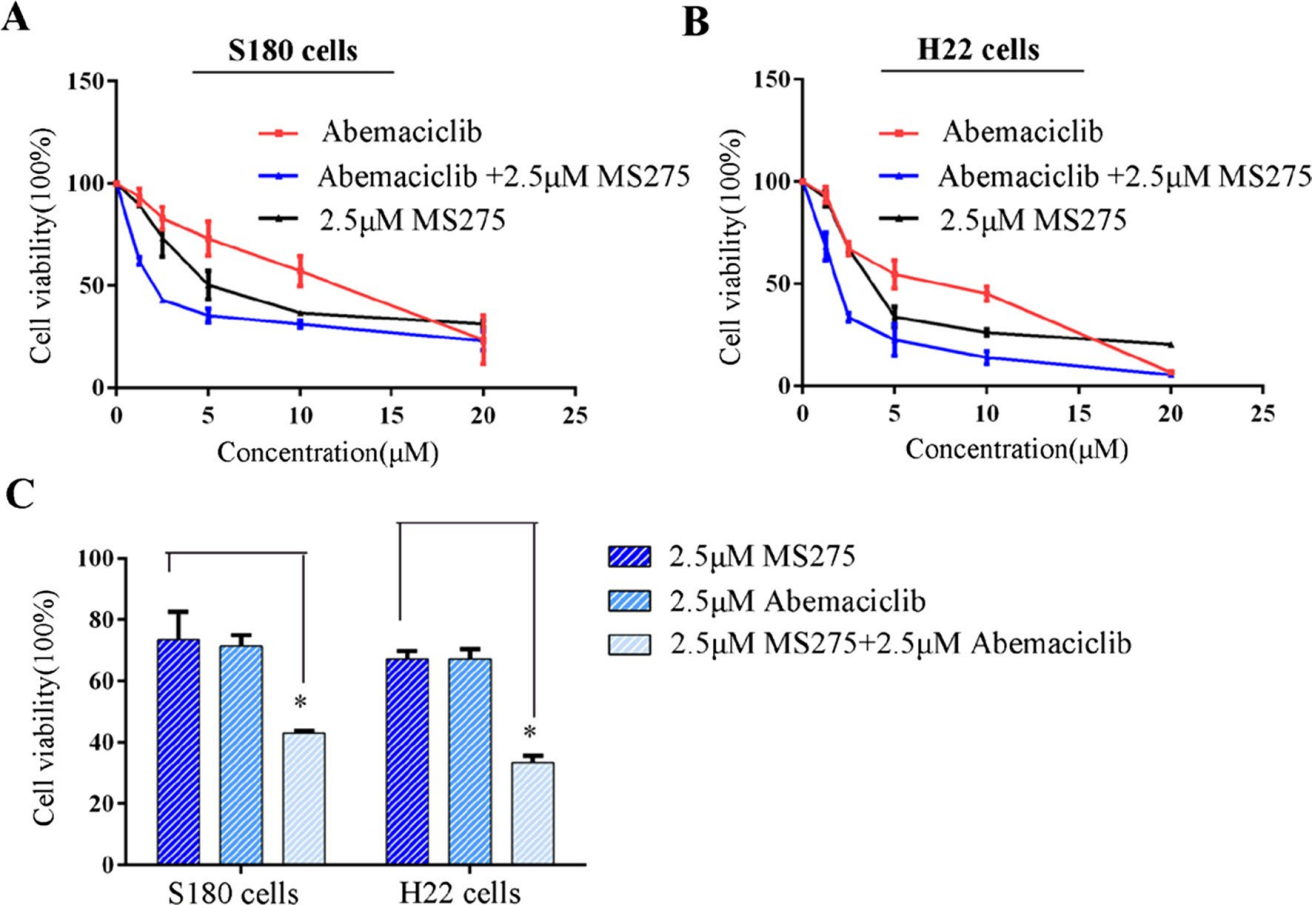
Viability of S180 and H22. S180 (**A**) and H22 (**B**) cells were incubated with 0 μM, 2.5 μM, 5 μM, 10 μM, 20 μM abemaciclib alone, or combination with 2.5 μM MS275 for 48 h. Each well contained 0.4% (v/v) DMSO. **C** Cell viability for S180, H22 cells when incubated with 2.5 μM MS275 alone, 2.5 μM abemaciclib alone, or combination of 2.5 μM MS275 and 2.5 μM abemaciclib for 48 h. Each well contained 0.4% (v/v) DMSO

Cyclin-dependent kinase 4 (CDK4) is a member of the cyclin-dependent kinase family, and can promote cell cycle progression. Through proteome analysis we found MS275 can downregulate CDK4 expression in S180 cells, which may somehow explain why MS275 had obvious effect on cell cycle arrest and inhibited the proliferation of malignant ascites cells. Then S180 cells or H22 cells were incubated with abemaciclib (CDK4/6 selective inhibitor) alone or in combination with MS275 shown in Fig. 5. Abemaciclib alone had an IC50 of 10.3 μM in S180 cells and 5.9 μM in H22 cells. Furthermore, we found when combination with 2.5 μM MS275, the IC50 decreased to 2.6 μM in S180 cells and 1.9 μM in H22 cells. Thus, Class I HDAC inhibitor MS275 and CDK4/6 inhibitor abemaciclib had synergistic anti-tumor effect and were more effective than MS275 or abemaciclib alone (Fig. 5C).

### MS275 inhibited ascites development, tumor growth, and prolonged survival time in mice

**Fig. 6:**
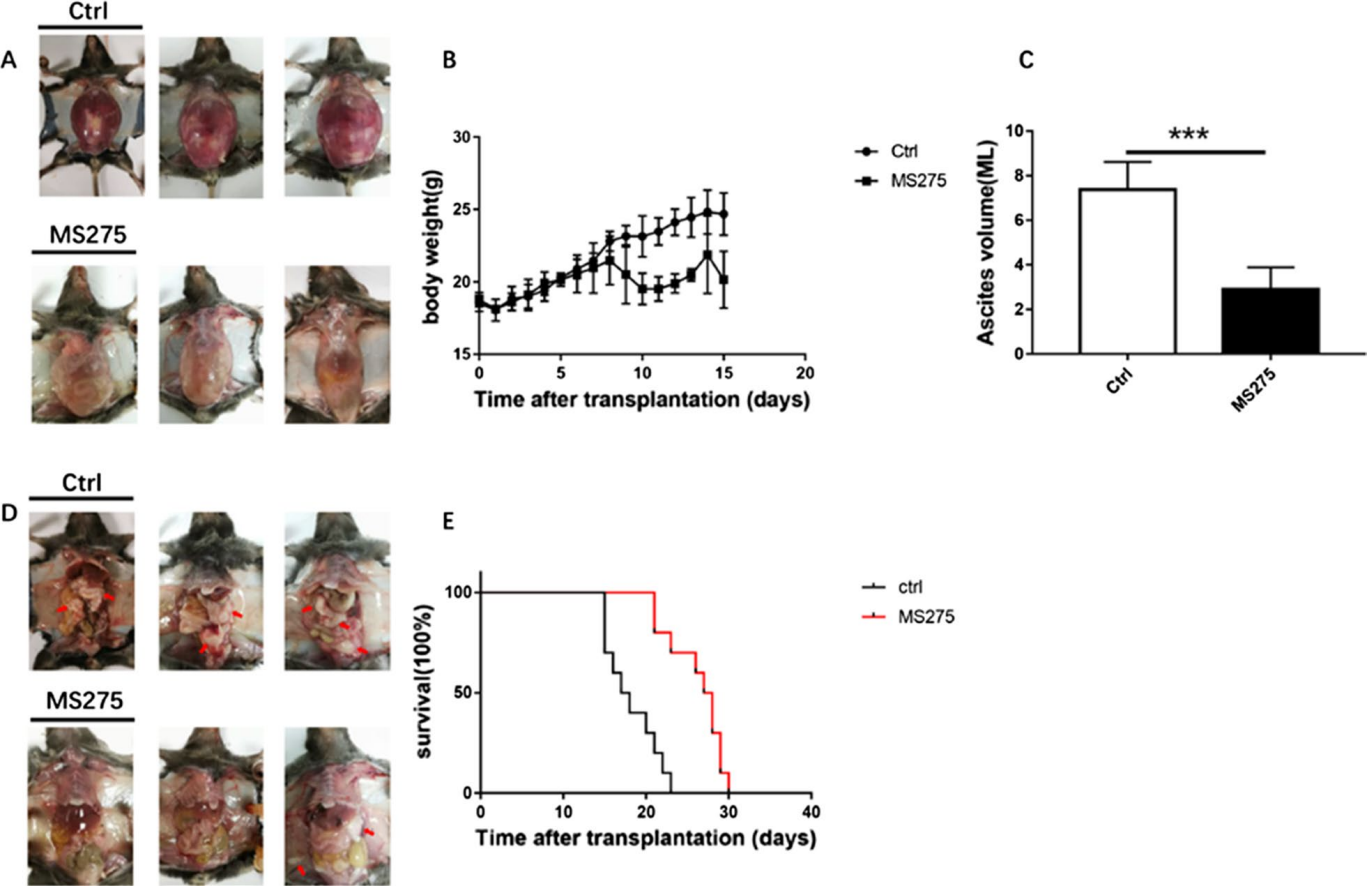
In vivo effect of MS275 on malignant ascites formation, tumor growth and mice survival. Compared with control group, less abdominal bulge (**A**) and parietal or visceral peritoneum carcinoma burden indicated with red arrows (**D**) were observed in MS275 treatment group. The body weight (**B**), the volume of ascites (**C**) at 24 h following the final treatment. Survival of mice after S180 cell inoculation into peritoneal cavity (n = 10) (**E**). **, *P* < 0.01 versus control group

Body weight was the direct index for determining the development of abdominal ascites tumors in mice [40]. To determine the effect of MS275 on malignant ascites formation and tumor growth, mice were administered with MS275 every other day for 4 consecutive intraperitoneal injection since the ascites models are formed. Comparing with the control group, the MS275 group seemed to have less abdominal bulge shown in Fig. 6A, and tumor burden shown in Fig. 6D. We tested the body weight of mice every day and found the MS275 group had lower body weight after 9 days’ treatment shown in Fig. 6B. The average ascites volume in the MS275 group was 2.9 ± 1.0 mL, much lower than 7.5 ± 1.2 mL in the control group shown in Fig. 6C, which indicated that ascites formation was significantly inhibited by MS275. In addition, the life-span increase of ascites-bearing mice in the MS275 treatment group was 66% when compared with the control group (Fig. 6E).

## Discussion

Malignant ascites can be caused by varieties of abdominal cancers, and associated with significant morbidity [1–4]. However, there is no generally accepted evidence-based guideline for the treatment of this disease [4], thus there is unmet demand to find new therapeutic approach for malignant ascites. In this paper, we found class I HDACI, MS275, exhibited preferential inhibition on different ascites cells, by inducing cell cycle arrest in G0/G1 phase and promoting apoptosis. Through proteome analysis, we found MS275 can regulate various signal pathways related to cell cycle, apoptosis, and tumorigenic pathways which might explain the underlying anti-tumor mechanism of MS275 in ascites cells. CDK4 was downregulated by MS275, we further observed that abemaciclib (CDK4/6 selective inhibitor) can inhibit the proliferation of ascites cells, and the combination of abemaciclib and MS275 exhibited better anticancer effect than the two drugs used alone. Finally, the in vivo mice model indicated that, MS275 could inhibit malignant ascites development, tumor growth, and prolong survival time.

HDACIs had been reported to have antiproliferative effects by inducing cell cycle arrest [41–44]. MS275 is a targeted HDAC1/3 inhibitors [45]. As reported, loss of HDAC1 induced expression of CDK inhibitors, leading to a cell cycle block in G1 in primary mouse fibroblasts and in the B-cell lineage [46]. Besides, HDAC1 knockdown in tumor cells could also impair G2/M transition and inhibits cell growth as evidenced by a reduction of mitotic cells [47]. In addition, HDAC3 knockdown caused cell accumulation in S and G2/M phases [48, 49]. We analyzed that the distribution difference of cell cycle arrest depends on HDAC inhibitor targets and cancer cell categories. In this study MS275 could induce cell cycle arrest in G0/G1 phase for S180 and H22 cells, and the downregulation of CDK4 pathway might be one of the explanation, which was consistent with previous study [44, 46]. Simultaneously our proteome analysis indicated that MS275 might affect cell cycle through other related proteins such as CDC20, EP300, CCND1, etc.

HDACs had been reported to regulate apoptosis in a variety of cancer cells through changing expression of pro- and antiapoptotic proteins [44]. Our data demonstrated that MS275 significantly promoted apoptosis for malignant ascites cells S180 and H22. Proteome data in our paper hinted MS275 could increase pro-apoptosis proteins expression such as PARP1, LMNB1, LMNB2, TR10B, ENSA, etc. In addition, MS275 could also affect cell growth and death through ferroptosis, necroptosis, cellular senescence through proteomic analysis. And the potential relations between HDACI and pro-apoptosis proteins found in our paper were rarely reported yet. The relevance of p53 in HDACI induced apoptosis was controversial [50], and MS275 activated p53 pathways in our data. Most studies pointed to a p53-independent action of HDACI because the anticancer effect of HDACI was not influenced by the tumor’s p53 status [51]. Other studies, however, suggested an essential role of p53 in the response of tumor cells to HDACI treatment [52]. Further exploration will be needed to test whether the pro-apoptosis effect of MS275 on S180 cell is dependent on P53 pathways or not.

Drug combination is a method to get better clinical outcome and less systemic toxicity. Proteome analysis in our study found abundant tumorigenic protein changes in MS275 treatment group, which may lay the foundation of drug combination for malignant ascites treatment. For example, CDK4 was downregulated by MS275 in proteomic analysis, we found here the combination of CDK4 inhibitor and MS275 had synergistic effect on MA cells. In the future more efficient therapy for MA might be found by combining MS275 and other tumorigenic protein inhibitors based on our proteome findings.

## Conclusions

In summary, this present research revealed that the class I selective HDACI, MS275, could effectively inhibit malignant ascites development and tumor growth. In the future, the initiation of clinical trials in humans with malignant ascites will be of interest.

## Data Availability

The proteomic data are accessible at the CNGB Sequence Archive (CNSA) of China National GeneBank DataBase (CNGBdb) with accession number CNP0002390. Other datasets used and/or analyzed are available from the corresponding author on reasonable request.

## Abbreviations

HDAC: Histone deacetylase
HDACI: Histone deacetylases inhibitor
CDK4: Cyclin-dependent kinase 4
MA: Malignant ascites
DMSO: Dimethyl sulfoxide
